# Supplementary material for: Identification of Prognostic Organic Cation and Anion Transporters in Different Cancer Entities by In Silico Analysis
Source: Int J Mol Sci. 2020 Jun 24;21(12):4491. doi: 10.3390/ijms21124491 (PMC7349956; doi:10.3390/ijms21124491)
Supplement: Supplementary file 1 [file ijms-21-04491-s001.zip › ijms-842918 suppplementary for proof.docx]

**Figure S1. Number of prognostic genes identified in the different tumor entities.** The red columns present the number of unfavorable and the green columns the number of favorable genes. Data derived from the Human Pathology Atlas [1].

**Figure S2.** Number of prognostic organic anion transporter organic anion transporter identified in the different tumor entities. The red columns present the number of unfavorable and the green columns the number of favorable genes. Data derived from the Human Pathology Atlas [1].

**Figure S3.** Intersection of prognostic organic anion transporter organic anion transporter identified in the different tumor entities. The list of favorable organic anion transporter organic anion transporter between the different tumor entities were compared and the number of common genes between a given comparison are highlighted in this figure. The intensity of the color indicates the number of common genes.

**Figure S4.** Intersection of prognostic unfavorable organic anion transporter organic anion transporter identified in the different tumor entities. The lists of unfavorable organic anion transporter organic anion transporter between the different tumor entities were compared and the number of common genes between a given comparisons are highlighted in this figure. The intensity of the color indicates the number of common genes.

**Figure S5**. Expression level of *SFXN2* and *UNC13b* in relation to treatment outcome in the TCGA PANCAN cohort. The expression level of *SFXN2* and *UNC13b* in patients classified for “treatment_outcome_first_course” with “Complete Remission/Response” or “Progressive Disease” from the TCGA PANCAN cohort are plotted here ^2^. T-test was used to identify statistically significant differences in expression level and are marked with * (*p* > 0.05).

**Figure S6.** Expression level of *PLS3,* *SLC2A1*, *SLC16A1* and *SLC16A3* in relation to treatment outcome in the TCGA PANCAN cohort. The expression level of *PLS3*, *SLC2A1, SLC16A1* and S*LC16A3* in patients classified for “treatment_outcome_first_course” with “Complete Remission/Response” or “Progressive Disease” from the TCGA PANCAN cohort are plotted here [2]. *t*-test was used to identify statistically significant differences in expression level and are marked by *(*p* > 0.05).

**References**

1. Uhlen, M.; Zhang, C.; Lee, S.; Sjostedt, E.; Fagerberg, L.; Bidkhori, G.; Benfeitas, R.; Arif, M.; Liu, Z.; Edfors, F.; Sanli, K.; von Feilitzen, K.; Oksvold, P.; Lundberg, E.; Hober, S.; Nilsson, P.; Mattsson, J.; Schwenk, J. M.; Brunnstrom, H.; Glimelius, B.; Sjoblom, T.; Edqvist, P. H.; Djureinovic, D.; Micke, P.; Lindskog, C.; Mardinoglu, A.; Ponten, F., A pathology atlas of the human cancer transcriptome. *Science* **2017,** *357* (6352).

2. Liu, J.; Lichtenberg, T.; Hoadley, K. A.; Poisson, L. M.; Lazar, A. J.; Cherniack, A. D.; Kovatich, A. J.; Benz, C. C.; Levine, D. A.; Lee, A. V.; Omberg, L.; Wolf, D. M.; Shriver, C. D.; Thorsson, V.; Cancer Genome Atlas Research, N.; Hu, H., An Integrated TCGA Pan-Cancer Clinical Data Resource to Drive High-Quality Survival Outcome Analytics. *Cell* **2018,** *173* (2), 400-416.e11.
